# Improving Recovery and Outcomes Every Day after the ICU (IMPROVE): study protocol for a randomized controlled trial

**DOI:** 10.1186/s13063-018-2569-8

**Published:** 2018-03-27

**Authors:** Sophia Wang, Jessica Hammes, Sikandar Khan, Sujuan Gao, Amanda Harrawood, Stephanie Martinez, Lyndsi Moser, Anthony Perkins, Frederick W. Unverzagt, Daniel O. Clark, Malaz Boustani, Babar Khan

**Affiliations:** 1 Department of Psychiatry, Indiana University School of Medicine, 355 W 16th Street, Suite 4800 GH, Room 4250, Indianapolis, IN 46202 USA; 2 Center of Health Innovation and Implementation Science, Center for Translational Science and Innovation, Indianapolis, IN USA; 3 Sandra Eskenazi Center for Brain Care Innovation, Eskenazi Hospital, Indianapolis, IN USA; 4 College of Arts and Sciences, Indiana University Bloomington, Bloomington, IN USA; 5 Division of Pulmonary, Critical Care, Sleep and Occupational Medicine, Department of Medicine, Indiana University School of Medicine, Indianapolis, IN USA; 6 Department of Biostatistics, Indiana University School of Medicine, Indianapolis, IN USA; 7 IU Center of Aging Research, Regenstrief Institute, Indianapolis, IN USA; 8 Division of Geriatrics and General Internal Medicine, Department of Internal Medicine, Indiana University School of Medicine, Indianapolis, IN USA

**Keywords:** Delirium, Physical exercise, Cognitive training, Critical care, Biomarkers, Alzheimer disease, Dementia, Aging, Post-intensive care syndrome, Internet delivery, Physical activity, Cognitive impairment

## Abstract

**Background:**

Delirium affects nearly 70% of older adults hospitalized in the intensive care unit (ICU), and many of those will be left with persistent cognitive impairment or dementia. There are no effective and scalable recovery models to remediate ICU-acquired cognitive impairment and its attendant elevated risk for dementia or Alzheimer disease (AD). The Improving Recovery and Outcomes Every Day after the ICU (IMPROVE) trial is an ongoing clinical trial which evaluates the efficacy of a combined physical exercise and cognitive training on cognitive function among ICU survivors 50 years and older who experienced delirium during an ICU stay. This article describes the study protocol for IMPROVE.

**Methods:**

IMPROVE is a four-arm, randomized controlled trial. Subjects will be randomized to one of four arms: cognitive training and physical exercise; cognitive control and physical exercise; cognitive training and physical exercise control; and cognitive control and physical exercise control. Facilitators administer the physical exercise and exercise control interventions in individual and small group formats by using Internet-enabled videoconference. Cognitive training and control interventions are also facilitator led using Posit Science, Inc. online modules delivered in individual and small group format directly into the participants’ homes. Subjects complete cognitive assessment, mood questionnaires, physical performance batteries, and quality of life scales at baseline, 3, and 6 months. Blood samples will also be taken at baseline and 3 months to measure pro-inflammatory cytokines and acute-phase reactants; neurotrophic factors; and markers of glial dysfunction and astrocyte activation.

**Discussion:**

This study is the first clinical trial to examine the efficacy of combined physical and cognitive exercise on cognitive function in older ICU survivors with delirium. The results will provide information about potential synergistic effects of a combined intervention on a range of outcomes and mechanisms of action.

**Trial registration:**

ClinicalTrials.gov, NCT03095417. Registered on 23 March 2017. Last updated on 15 May 2017.

**Electronic supplementary material:**

The online version of this article (10.1186/s13063-018-2569-8) contains supplementary material, which is available to authorized users.

## Background

Delirium is defined as acute brain dysfunction characterized by an inability to focus, sustain, or shift attention that develops quickly and tends to fluctuate throughout the day [1]. Nearly 70% of older adults who become critically ill and require hospitalization in the intensive care unit (ICU) develop delirium. ICU delirium can lead to immediate in-hospital complications including a longer length of ICU and hospital stay, increased risk of in-patient mortality, and elevated costs of care. In addition, ICU delirium is associated with long-term post-discharge complications, such as the development of cognitive impairment and dementia [2–5].

**Fig. 1:**
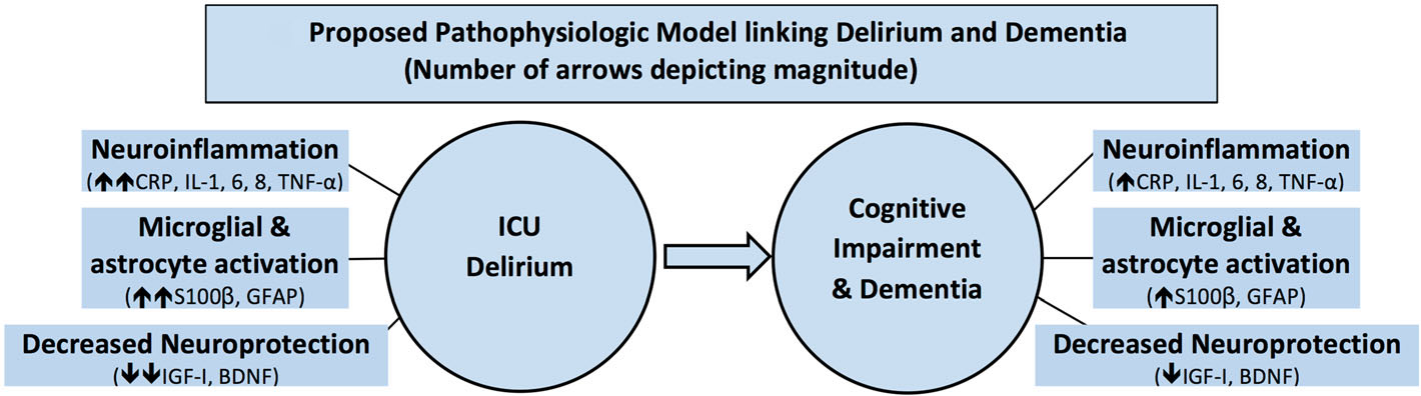
Proposed pathophysiologic model linking delirium and dementia (*number of arrows* depicting magnitude)

Delirium can be considered a cytokine-mediated inflammatory syndrome. The cytokine-mediated systemic inflammation eventually results in microglial and astrocyte activation, neuroinflammation, and lower levels of neuroprotection [6–8]. The combination of these three factors can increase the risk of delirium. These biochemical changes associated with delirium can then persist, albeit at a lower level, and may result in a chronic state of neuroinflammation, neurotoxicity, diminished neuroplasticity, and cholinergic failure, which then eventually manifests as long-term ICU-acquired cognitive impairment, dementia, or Alzheimer disease (AD, see Fig. 1).

Physical activity has been found to enhance angiogenesis, neurogenesis, the release of neurotrophic factors, and neuroplasticity in animal studies [9–11]. Exercise has also been found to increase cerebral blood flow, oxygen extraction and glucose use, and reduce inflammation [12, 13]. Older patients who perform aerobic exercise showed the greatest increase in blood volume in frontal and parietal lobe white matter [14–18]. Executive cognitive ability showed the largest response to exercise as compared to other cognitive domains. Gains were also greater for training that lasted 30–45 min (versus longer or shorter) and older subjects (versus ones that were age 55–65) [19–25]. Physical activity also improves cognition in well older adults, and those with mild cognitive impairment, AD, stroke, chronic obstructive pulmonary disease (COPD), and traumatic brain injury [19–25].

Cognitive training has also been demonstrated to have favorable cognitive effects. The largest and most rigorous investigation of the efficacy of cognitive training is the Advanced Cognitive Training for Independent and Vital Elderly (ACTIVE) trial. ACTIVE randomized 2802 well-functioning, community-dwelling adults (65 years and older) to Memory, Reasoning, Speed of Processing training, or a no-contact control group [26]. Each ACTIVE intervention produced an immediate improvement in the trained ability with largest improvements observed for the Speed of Processing intervention followed by Reasoning and Memory. Treatment effects were maintained for 5 years on Memory, Reasoning, and Speed trained participants relative to controls and up to 10 years for Reasoning and Speed. A recent meta-analysis of trials of cognitive training on persons with MCI also found mild to moderate cognitive benefits from cognitive training although the long-term effects are not clear [27].

The biochemical pathways through which physical and cognitive exercise can improve cognition are still being characterized. Physical exercise appears to mitigate endothelial dysfunction and vascular wall inflammation, and enhances neural substrates brain-derived neurotrophic factor (BDNF) and insulin growth factor-1 (IGF-1) that maximize the effects of subsequent cognitive stimulation [28, 29]. Similarly, cognitive therapy may exert its favorable effect by increasing levels of BDNF. This was shown in a randomized trial of 8 weeks of cognitive therapy versus health education among patients with heart failure [30]. Patients randomized into the cognitive therapy arm showed an increase in their BDNF levels at 8 weeks compared to baseline levels as well as improvement in working memory, whereas the BDNF levels decreased in the education control.

**Fig. 2:**
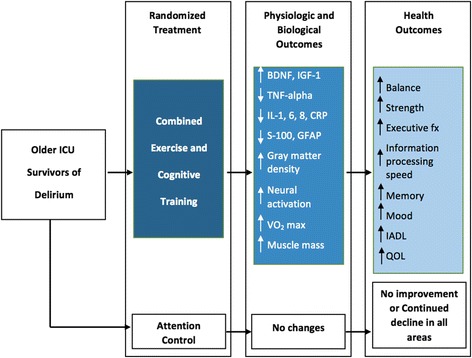
Conceptual model of the IMPROVE study

Based on the prior work and the pathophysiologic mechanisms described above, the Improving Recovery and Outcomes Every Day after the ICU (IMPROVE) clinical trial was developed to test a novel home-based combined physical exercise and cognitive training program for older ICU survivors with delirium to improve cognitive impairment. Figure 2 describes the conceptual model behind the study. In this model, physical exercise has an inducing effect on neural proliferation while cognitive training promotes the long-term functional integration of these new neural elements into adaptive networks [9, 10]. Thus, the enhancement in neural function and cognitive capacity produced by the combined intervention is hypothesized to result in improvement in ICU-acquired cognitive disability, and ultimately a delay in the time-to-onset of Alzheimer disease and related dementias. Improvements in physical and behavioral performance will be reflected as improved insulin resistance, VO2 max, muscle mass, and cognitive ability and mood. These cascades of physiological and behavioral changes result in very broad levels of improved cognitive performance, mood symptoms, functional independence, and quality of life.

Based on the above findings, we hypothesize that combined physical exercise and cognitive training will produce improved biologic and physiologic substrates with increased BDNF and IGF-1 leading to neural proliferation and improved inflammatory environment [decrease in tumor necrosis factor (TNF)-α, interleukin (IL)-1, 6, 8, and C-reactive protein (CRP)]. These changes in turn will promote improvements to brain structure (increased frontal and temporal gray matter density) and neural activation, and reduce astrocyte and microglial activation [decrease in S-100β and glial fibrillary acidic protein (GFAP)].

The primary outcome of IMPROVE is to determine the effects of the combined physical exercise and cognitive training on the cognitive function of ICU survivors aged 50 and older*.* The secondary outcomes of IMPROVE are to determine the effects of the combined physical exercise and cognitive training on physical performance, anxiety and depressive symptoms, and quality of life of this patient population, and to examine the mechanisms of action of combined training by measuring biomarkers relevant to delirium and dementia [6, 31, 32].

## Methods/Design

### Study setting and design

**Fig. 3:**
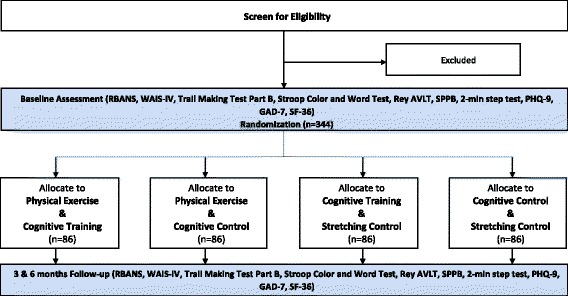
Eligibility, randomization, allocation, and follow-up outcomes

This protocol is described as required by the 2013 SPIRIT guidelines to ensure consistent reporting of clinical trials (see Additional file 1). Study design is depicted in Fig. 3. Subjects will be randomized to one of four arms: cognitive control and physical exercise control; cognitive control and physical exercise; cognitive training and physical exercise control; and cognitive training and physical exercise. A total of 344 subjects will be enrolled. 86 subjects will be randomized to each arm.

The target population for the IMPROVE trial is adults aged 50 and older who have survived a critical illness in the ICU and had a delirium episode during their ICU stay at any of the three Indiana University School of Medicine-affiliated hospitals; IU Health (IUH) Methodist Hospital; IUH University Hospital; and Eskenazi Hospital. Trained research assistants will screen eligible individuals (those who meet inclusion criteria and do not meet any exclusion criteria) who will be screened twice per day for delirium until ICU discharge using the Confusion Assessment Method for the ICU (CAM-ICU) [33]. Patients who screened positive on the CAM-ICU and survived the ICU stay will be approached for enrollment into the study within 48 h of their anticipated hospital discharge.

**Fig. 4:**
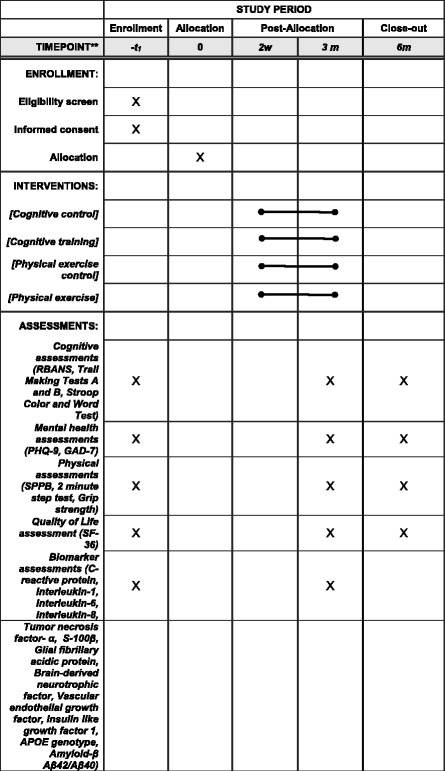
Schedule of enrollment, interventions, and assessments. GAD-7 Generalized Anxiety Disorder-7; PHQ-9 Patient Health Questionnaire-9; RBANS Repeatable Assessment of Neuropsychological Status; SF-36 36-Item Short Form Health Survey; SPPB Short Physical Performance Battery

Study staff will complete a baseline assessment consisting of measures of cognition, physical function, depression and anxiety, and quality of life within 2 weeks of hospital discharge (Figs. 3 and 4). Blood samples will also be collected from the participants at the time of baseline assessment. After the initial assessment, study subjects will be randomized to study groups. Randomization will be stratified by age (50-64, 65–75, older than 75), and by hospital site (Eskenazi, University, Methodist), for a total of 9 strata. A computer-generated randomization within stratum will be done using random blocks of 8 or 12, stratifying by discharged home versus discharged other (80% randomization block size 4 : 20% randomization block size 8). The risk of patient withdrawal will be addressed through the following approaches: availability of make-up sessions, availability of individual training sessions, study staff follow-up with the study participant if participation is less than 80%, and gift certificate incentives for completion of 3 and 6 months follow-up assessments.

Outcomes assessments will be conducted by research assistants who are blinded to randomization assignment at 3 and 6 months post baseline. Outcomes assessments consist of measures of cognition, physical function, depression and anxiety, and quality of life (see Outcome measures section in Fig. 4). Data will be collected and stored using Research Electronic Data Capture (REDCap). Research assistants will undergo training and certification procedures with a supervisor for quality assurance. Ongoing quality assurance checks are performed by the supervisor at regular intervals. Subjects will be instructed not to discuss their assigned intervention with the research assistants.

### Participants

Participants must meet all the inclusion criteria below:

1. Age ≥ 50 years
2. Admitted to medical or surgical ICU at Methodist, University, or Eskenazi hospitals
3. English-speaking
4. Discharged home or subacute rehabilitation
5. Able to provide consent or has a legally authorized representative to provide consent
6. Access to a telephone
7. Have at least one episode of delirium as determined by the Confusion Assessment Method in the ICU

Participants who meet any of the following criteria will be excluded:

1. Diagnosis of cancer with short life expectancy
2. Current chemotherapy or radiation therapy
3. History of dementing illnesses and other neurodegenerative diseases such as Alzheimer disease, Parkinson disease, or vascular dementia
4. Current alcohol consumption ≥ 5 drinks per day
5. Vision < 20/80 via Snellen card
6. Low hearing or communicative ability (examiner rated) that would interfere with interventions and outcome assessments
7. Presence of delirium at time of hospital discharge
8. Presence of American College of Sports Medicine absolute or relative contraindications to exercise
9. Recovering from a skeletal fracture
10. Stroke as the admitting diagnosis or a new event during the course of hospitalization
11. Recent history of drug abuse [Drug Abuse Screening Test (DAST-20) score > 5]

### Interventions

Enrolled subjects randomized to the active cognitive intervention will receive facilitator-led cognitive training via computer-accessed online training modules 45 min per session, 2 days per week for 3 months; and physical exercise delivered by trained facilitators to participants in their homes via Internet-based single or multi-party (2–6 per group) videoconference 45 min per session, three times per week for 3 months.

The active cognitive intervention consists of several modules from Brain HQ developed by Posit Science, Inc., that engage time-order judgment, visual discrimination, spatial-match, forward-span, instruction-following, dual task, and memory. Exercises adapt to participants’ level of performance and automatically advance in difficulty level as performance improves.

The cognitive control intervention consists of control modules also from Brain HQ. The cognitive control activities happen at the same frequency as the active cognitive intervention.

The active physical exercise intervention consists of 45 min of multi-modal physical exercise focused on seated aerobic and progressive resistance training designed to improve aerobic capacity, muscular strength and endurance consistent with current exercise recommendations. Three sessions per week for 3 months will be delivered. Participant’s heart rate and rating of perceived exertion (RPE) will be used to ascertain achievement of moderate intensity exercise. Participants will be advised to exercise at the RPE level of 5–6 on a 10-point scale, equating to moderate-intensity exercise for adults over the age of 40 years. Each physical exercise session will be divided as follows: 5 min of warm-up, 10 min of upper-body, 10 min of core, 10 min lower-body, 5 min of upper-body and lower-body, and 5 min of cool-down and flexibility exercises. All exercises will be conducted from a seated position in a solid backed chair.

The physical exercise control will consist of gentle, seated stretching. Three sessions per week for 3 months will be delivered.

### Outcome measures

The primary outcome will be cognitive performance at 3 and 6 months. The Repeatable Battery for the Assessment of Neuropsychological Status (RBANS) total index score will provide the primary outcome for the trial [34]. Secondary outcomes will be assessed using individual tests of processing speed, executive control, and new learning ability as follows: Trail Making Test Part A and B (seconds to complete), and Stroop Color and Word Test (interference trial). These measures sample major domains of cognition affected in ICU survivors.

Secondary outcomes will also include mood symptoms, physical performance, quality of life, and biomarkers relevant to delirium and dementia. All secondary outcomes, except for biomarkers, will be measured at 3 and 6 months. Mood symptoms will be assessed with Patient Health Questionnaire-9 (PHQ-9) and Generalized Anxiety Disorder Scale-7 (GAD-7) to determine the impact of the intervention on the ICU’s survivors’ mood and anxiety. The PHQ-9 is a nine-item depression scale with a total score from 0 to 27 [35, 36] and the GAD-7 is a seven-item anxiety scale with a total score from 0 to 21 [37, 38].

Physical training effects on balance and strength will be assessed via the Short Physical Performance Battery (SPPB), a validated objective assessment [39, 40]. The SPPB yields a performance score of 0–12 (0–4 poor, 5–7 intermediate, 8–12 good). A difference of 1 point indicates a significant change in function. Physical training effects on cardiovascular fitness will be assessed via the 2-min step test and grip strength. The 2-min step test for cardiovascular fitness is a validated measure of aerobic capacity, does not require equipment, and can be used in the home setting. Grip strength will be measured using a JAMAR hand dynamometer [41].

The Medical Outcome Study Short Form (SF-36) will be used to measure quality of life. It has eight components (physical functioning, role-physical, bodily pain, general health, vitality, social functioning, role-emotional, and mental health) that are aggregated into a Physical Component Summary (PCS) and a Mental Component Summary (MCS). The PCS and the MCS will be used as quality of life outcomes.

Biomarkers will be measured at baseline and 3 months: pro-inflammatory cytokines (IL-1, 6, 8, TNF-α); the acute-phase reactant (CRP); neurotrophic factors [IGF-1, vascular endothelial growth factor (VEGF), BDNF]; markers of glial dysfunction and astrocyte activation (S-100β, GFAP); and AD-related biomarkers (*APOE* genotype, plasma amyloid-β Aβ42/Aβ40) [31, 32].

### Statistical analyses

Statistical analyses will be conducted using SAS 9.4 (SAS Institute, Cary, NC, USA). Differences in patients’ baseline characteristics among the four groups will be compared using analysis of covariance (ANCOVA) for continuous variables and the Cochran-Mantel-Hansel statistic for categorical variables while adjusting for stratification variables.

Mixed effects models will be used with repeated RBANS scores collected at baseline, 3 months, and 6 months as the outcome measures, group, time, and a group by time interaction as independent variables while adjusting for stratification variables and other potential baseline covariates found to be significantly different in univariate comparisons. A significant interaction between group and time would indicate differences in changes of cognitive functions over time among the four groups. Post hoc comparisons will be conducted following a significant interaction between group and time to compare the effect of the combined training group to the other three groups (attention control, cognitive training only, and exercise only groups) at 3 month for immediate training effect and at 6 months for sustained training effect. Separate mixed-effect models will also be used for repeated Trail Making Test Part A and B and Stroop Color and Word Test.

Similarly, separate mixed-effects models will be used with repeated measures (SPPB, 2-min step, PHQ-9, GAD-7, SF-36 PCS and MCS) as the outcome variables, group, time, and interactions between group and time as independent variables, while adjusting for stratification variables and other baseline covariates that may be different among the four groups. Significant interactions between group and time in these models would indicate differences in changes of functional outcomes, depressive symptoms, and anxiety levels or quality of life over time among the four groups. Post hoc analyses will also be conducted following significant interactions in the mixed-effects models to compare the combined training group to the other three groups and to determine how early a group difference can be detected and whether the effect extends beyond the training period.

Changes in the serum levels of CRP, IL-1, IL-6, IL-8, TNF-α, S-100β, GFAP, BDNF, VEGF, and IGF-1, and plasma levels of amyloid beta Aβ42/Aβ40 will be calculated and used as the dependent variables in ANCOVA models with group as the independent variable and adjusting for stratification variables and baseline covariates that are found to be different among the groups in univariate comparisons. Post hoc comparisons will be used following a significant group effect to compare biomarker levels in the combined training group to the other three groups.

Missing data are expected due to patient death (mostly within 30 days of ICU discharge) and withdrawal from treatment. Patient death rates are expected to be equal across all four groups. The mixed-effects model approach is robust under the missing at random assumption, i.e. the probability of missing is unrelated to the missing observations. Baseline characteristics of subjects with missing outcomes due to death or dropout during follow-up will be compared to detect potential violation to the missing at random assumption. Intention to treat analysis will be used in all models. Further sensitivity analyses will be performed using various methods of imputation or a full parametric likelihood approach assuming various patterns of missing data [42].

Sample size calculation was performed as follows. For the primary outcome to measure differences in cognitive effects, cognitive training has been found to have a moderate effect size of approximately 0.5 SD and the combined training was found to have a larger effect size of 0.9 SD when comparing to the control group in healthy elderly subjects [43–45]. Assuming effect sizes of 0.4 SD in the cognitive training only group and the exercise-only groups at 3 and 6 months post baseline compared to the attention control group and effect size of 0.8 SD in the combined training group at 3 and 6 months post baseline compared to the control group, a sample size of 60 patients in each group will yield 83% power at detecting a significant group by time interaction in a mixed-effects model adjusting for correlations of 0.2 for outcomes measured 3 months apart, and correlations of 0.1 for outcomes measured 6 months apart at α = 0.05. The power estimation was conducted using the GLMPower procedure in SAS. To further assume that 30% patients may miss some post-baseline assessments, we will need to enroll a total of 344 patients into the study (86 patients per group).

The proposed analyses for the outcomes for the mood questionnaires, physical performance battery, and quality of life scales will also have 83% power to detect effect sizes similar to those described for the primary outcome about cognitive effects. For the proposed analyses in changes in biomarkers, there will be 81.7% power to detect an overall treatment effect with effect sizes of 0.31 SD in the cognitive training only and the exercise only groups and 0.62 SD in the combined training groups at α = 0.05 using one-way ANOVA and the Power procedure in SAS.

### Monitoring

The exercise program elements will include warm-up, flexibility exercise, moderate intensity exercise, and a gradual progression of exercise intensity and duration, and cool down. These elements are associated with lower risks of cardiovascular complications and muscular injury. The Interventionist will educate subjects about the signs and symptoms of angina, myocardial infarction, and muscle/tendon-related injuries and how to respond to them; a procedure recommended by the American College of Sports Medicine and the American Heart Association as effective means of reducing complications to exercise [46, 47]. The Data and Safety Monitoring Plan includes the identification and response to serious cardiac events and orthopedic injuries in the Protection of Human Subjects section. Interventionists will be alert to signs of anxiety and frustration displayed by subjects during cognitive training. Interventionists will monitor reactions closely and respond appropriately with encouragement and suggestions to reduce burden.

## Discussion

Despite the high prevalence of post-ICU cognitive impairment and dementia, there are no effective and scalable recovery models to remediate these complications. IMPROVE represents a major step forward in the field of post-ICU cognitive impairment by proposing a novel home-based combined physical exercise and cognitive training program. The approach to deliver these trainings through the Internet increases the feasibility of participation and has the potential for widespread dissemination of this modality. The design of IMPROVE is based on a combination of previous knowledge about the effect of cognitive and physical training on cognitive outcomes and older adults, and the underlying pathophysiology of long-term cognitive impairment due to ICU delirium.

There are, however, limitations related to the sample. Previous studies suggest the majority of deaths and hospital readmission occur within the first 30 days of ICU discharge [48, 49]. More than 15% of ICU patients are hospitalized within 30 days of discharge [49]. Likewise, the 30-day mortality rate for adult ICU patients is also higher than 15% [48]. Exclusion criteria have been designed to increase the likelihood of selecting participants with at least a 6-month life expectancy. Assistive devices (glasses, headphones or pocket talker) will also be provided during interventions and assessments when indicated.

Despite these limitations, IMPROVE is innovative in several aspects. First, this is the first clinical trial in ICU survivors of delirium to examine the efficacy of interventions in isolation (physical exercise versus cognitive training versus attention control) or in combination (physical exercise plus cognitive training versus attention control). Second, this proposed intervention is based on plausible pathophysiologic pathways. The collection of biomarkers will help in establishing the mechanisms between cognitive activity, physical activity, and cognitive decline among ICU survivors of delirium. Third, this approach of using a combined physical exercise and cognitive training is an innovative behavioral intervention that has the potential for additive effects on enhancing cognitive recovery following critical illness. Finally, the proposed use of computers and broadband Internet to deliver the physical exercise and cognitive training to older ICU survivors in their homes using trained coaches via videoconference reduces task difficulty and is part of the growing movement championed by the Centers for Disease Control called Healthy Aging 2.0 which encourages health service delivery via the Internet [50].

In summary, IMPROVE is the first randomized controlled trial to evaluate the efficacy of combined cognitive and physical training in improving the cognitive function among ICU survivors 50 or older who experienced delirium during their ICU stay. It will also provide valuable biomarker data to more deeply understand the pathophysiology of long-term cognitive impairment and dementia in survivors of ICU delirium, and potential therapeutic targets for future studies. Most importantly, this study will pave the way for a large effectiveness randomized controlled trial to test the combined intervention to delay time to a clinical diagnosis of Alzheimer disease and related dementias.

## Trial status

The study started recruiting October 1, 2017. The expected completion date is June 1, 2023.

## Additional file

Additional file 1: SPIRIT 2013 Checklist: Recommended items to address in a clinical trial protocol and related documents*. (DOCX 41 kb)

## Abbreviations

ACTIVE: Advanced Cognitive Training for Independent and Vital Elderly
AD: Alzheimer disease
ANCOVA: Analysis of covariance
BDNF: Brain-derived neurotrophic factor
CAM-ICU: Confusion Assessment Method for the ICU
COPD: Chronic obstructive pulmonary disease
CRP: C-reactive protein
DAST-20: Drug Abuse Screening Test
GAD-7: Generalized Anxiety Disorder Scale
GFAP: Glial fibrillary acidic protein
ICU: Intensive care unit
IGF-1: Insulin growth factor-1
IL: Interleukin
IMPROVE: Improving Recovery and Outcomes Every Day after the ICU
MCS: Mental Component Summary
PCS: Physical Component Summary
PHQ-9: Patient Health Questionnaire-9
RBANS: Repeatable Assessment of Neuropsychological Status
RPE: Rating of perceived exertion
SD: Standard deviation
SF-36: 36-Item Short Form Health Survey
SPPB: Short Physical Performance Battery
TNF: Tumor necrosis factor
VEGF: Vascular endothelial growth factor

## References

[1] American Psychiatric Association (2013). Diagnostic and statistical manual of mental disorders: DSM-5.

[2] Khan BA, Lasiter S, Boustani MA (2015). Critical Care Recovery Center. Making the case for an innovative collaborative care model for ICU survivors. Am J Nurs..

[3] Pandharipande PP, Girard TD, Jackson JC (2013). Long-term cognitive impairment after critical illness. N Engl J Med..

[4] Girard TD, Jackson JC, Pandharipande PP (2010). Delirium as a predictor of long-term cognitive impairment in survivors of critical illness. Crit Care Med..

[5] Guerra C, Linde-Zwirble WT, Wunsch H (2012). Risk factors for dementia after critical illness in elderly Medicare beneficiaries. Crit Care..

[6] Hofer S, Bopp C, Hoerner C (2008). Injury of the blood brain barrier and up-regulation of ICAM-1 in polymicrobial sepsis. J Surg Res..

[7] Nishioku T, Sohgu S, Takata F (2009). Detachment of brain pericytes from the basal lamina is involved in disruption of the blood-brain barrier caused by lipopolysaccharide-induced sepsis in mice. Cell Mol Neurobiol..

[8] Semmler A, Okulla T, Sastre M, Dumitrescu-Ozimek L, Heneka M (2005). Systemic inflammation induces apoptosis with variable vulnerability of different brain regions. J Chem Neuroanat..

[9] Kempermann G, Fabel K, Ehninger D (2010). Why and how physical activity promotes experience-induced brain plasticity. Front Neurosci..

[10] Erickson KI, Kramer AF (2009). Aerobic exercise effects on cognitive and neural plasticity in older adults. Br J Sports Med..

[11] Fabel K, Wolf SA, Ehninger D, Babu H, Leal-Galicia P, Kempermann G (2009). Additive effects of physical exercise and environmental enrichment on adult hippocampal neurogenesis in mice. Front Neurosci..

[12] Churchill JD, Galvez R, Colcombe S, Swain RA, Kramer AF, Greenough WT (2002). Exercise, experience and the aging brain. Neurobiol Aging..

[13] Gleeson M, Bishop NC, Stensel DJ (2011). The anti-inflammatory effects of exercise: mechanisms and implications for the prevention and treatment of disease. Nat Rev Immunol..

[14] Colcombe SJ, Erickson KI, Scalf PE, Kim JS, Prakash R, McAuley E, Elavsky S, Marquez DX, Hu L, Kramer AF (2006). Aerobic exercise training increases brain volume in aging humans. J Gerontol A Biol Sci Med Sci..

[15] Erickson KI, Prakash RS, Voss MW, Chaddock L, Hu L, Morris KS, White SM, Wojcicki TR, McAuley E, Kramer AF (2009). Aerobic fitness is associated with hippocampal volume in elderly humans. Hippocampus..

[16] Erickson KI, Raji CA, Lopez OL, Becker JT, Rosano C, Newman AB, Gach HM, Thompson PM, Ho AJ, Kuller LH (2010). Physical activity predicts gray matter volume in late adulthood: the Cardiovascular Health Study. Neurology..

[17] Marks BL, Katz LM, Styner M, Smith JK (2011). Aerobic fitness and obesity: relationship to cerebral white matter integrity in the brain of active and sedentary older adults. Br J Sports Med..

[18] Marks BL, Madden DJ, Bucur B, Provenzale JM, White LE, Cabeza R, Huettel SA (2007). Role of aerobic fitness and aging on cerebral white matter integrity. Ann N Y Acad Sci..

[19] Laurin D, Verreault R, Lindsay J, MacPherson K, Rockwood K (2001). Physical activity and risk of cognitive impairment and dementia in elderly persons. Arch Neurol..

[20] Palleschi L, Vetta F, De Gennaro E, Idone G, Sottosanti G, Gianni W, Marigliano V (1996). Effect of aerobic training on the cognitive performance of elderly patients with senile dementia of Alzheimer type. Arch Gerontol Geriatr..

[21] Pyoria O, Talvitie U, Nyrkko H, Kautiainen H, Pohjolainen T, Kasper V (2007). The effect of two physiotherapy approaches on physical and cog- nitive functions and independent coping at home in stroke rehabilitation. A preliminary follow-up study. Disabil Rehabil..

[22] Quaney BM, Boyd LA, McDowd JM, Zahner LH, He J, Mayo MS, Macko RF (2009). Aerobic exercise improves cognition and motor function poststroke. Neurorehabil Neural Repair..

[23] Ozdemir F, Birtane M, Tabatabaei R, Kokino S, Ekuklu G (2001). Comparing stroke rehabilitation outcomes between acute inpatient and non-intense home settings. Arch Phys Med Rehabil..

[24] Etnier JL, Berry M (2001). Fluid intelligence in an older COPD sample after short- or long-term exercise. Med Sci Sports Exerc..

[25] Grealy MA, Johnson DA, Rushton SK (1999). Improving cognitive function after brain injury: the use of exercise and virtual reality. Arch Phys Med Rehabil..

[26] Unverzagt FW, Kasten L, Johnson KE (2007). Effect of memory impairment on training outcomes in ACTIVE. J Int Neuropsychol Soc..

[27] Li HJ (2011). Cognitive intervention for persons with mild cognitive impairment: a meta-analysis. Ageing Res Rev..

[28] Voss MW, Nagamatsu LS, Liu-Ambrose T, Kramer AF (2011). Exercise, brain, and cognition across the life span. J Appl Physiol..

[29] Ribeiro F, Alves AJ, Duarte JA, Oliveira J (2010). Is exercise training an effective therapy targeting endothelial dysfunction and vascular wall inflammation?. Int J Cardiol..

[30] Pressler SJ, Titler M, Koelling TM (2015). Nurse-enhanced computerized cognitive training increases serum brain-derived neurotropic factor levels and improves working memory in heart failure. J Card Fail..

[31] Khan BA, Farber MO, Campbell NL (2013). S-100 calcium binding protein as a biomarker for delirium duration in the intensive care unit. An exploratory analysis. IJGM..

[32] Khan B, Zawahiri M, Campbell N, Boustani M (2011). Biomarkers for delirium - a review. J Am Geriatr Soc..

[33] Ely EW, Margolin R, Francis J (2001). Evaluation of delirium in critically ill patients: validation of the Confusion Assessment Method for the Intensive Care Unit (CAM-ICU). Crit Care Med..

[34] Randolph C, Tierney MC, Mohr E (1998). The repeatable battery for the assessment of neuropsychological status (RBANS): preliminary clinical validity. J Clin Exp Neuropsychol..

[35] Kroenke K, Spitzer RL, Williams JB (2001). The PHQ-9: validity of a brief depression severity measure. J Gen Intern Med..

[36] Lowe B, Unutzer J, Callahan CM, Perkins AJ, Kroenke K (2004). Monitoring depression treatment outcomes with the patient health questionnaire-9. Med Care..

[37] Kroenke K, Spitzer RL, Williams JB, Monahan PO, Lowe B (2007). Anxiety disorders in primary care: prevalence, impairment, comorbidity, and detection. Ann Intern Med..

[38] Spitzer RL, Kroenke K, Williams JB, Lowe B (2006). A brief measure for assessing generalized anxiety disorder: the GAD-7. Arch Intern Med..

[39] Guralnik JM, Ferrucci L, Pieper CF (2000). Lower extremity function and subsequent disability: consistency across studies, predictive models, and value of gait speed alone compared with the short physical performance battery. J Gerontol A Biol Sci Med Sci..

[40] Rikli R, Jones CJ (2001). American College of Sports Medicine: Senior fitness test manual.

[41] Lafayette Instrument (2004). JAMAR Hydrolic hand dynamometer user instructions.

[42] Little RJA, Robin DB (2002). Statistical analysis with missing data.

[43] Fabre C (2002). Improvement of cognitive function by mental and/or individualized aerobic training in healthy elderly subjects. Int J Sports Med..

[44] Oswald WD (2006). Differential effects of single versus combined cognitive and physical training with older adults: The SimA study in a 5-year perspective. Eur J Ageing..

[45] Valenzuela M, Sachdev P (2009). Can cognitive exercise prevent the onset of dementia? Systematic review of randomized clinical trials with longitudinal follow-up. Am J Geriatr Psychiatry..

[46] Garber CE (2011). Quantity and quality of exercise for developing and maintaining cardiorespiratory, musculoskeletal, and neuromotor fitness in apparently healthy adults: guidance for prescribing exercise. Med Sci Sports Exerc..

[47] Thompson PD (2007). Exercise and acute cardiovascular events placing the risks into perspective: a scientific statement from the American Heart Association council on Nutrition, Physical Activity, and Metabolism and the Council on Clinical Cardiology. Circulation..

[48] Hua M, Gong MN, Brady J, Wunsch H (2015). Early and late unplanned rehospitalizations for survivors of critical illness. Crit Care Med..

[49] Kim MM, Barnato AE, Angus DC, Fleisher LA, Kahn JM (2010). The effect of multidisciplinary care teams on intensive care unit mortality. Arch Intern Med..

[50] Hall AK, Stellefson M, Bernhardt JM (2012). Healthy aging 2.0: the potential of new media and technology. Prev Chronic Dis..

