# Supplementary material for: Improving Recovery and Outcomes Every Day after the ICU (IMPROVE): study protocol for a randomized controlled trial
Source: Trials. 2018 Mar 27;19:196. doi: 10.1186/s13063-018-2569-8 (PMC5869765; doi:10.1186/s13063-018-2569-8)
Supplement: Supplementary file 1 — SPIRIT 2013 Checklist: Recommended items to address in a clinical trial protocol and related documents*. (DOCX 41 kb) [file 13063_2018_2569_MOESM1_ESM.docx]

SPIRIT 2013 Checklist: Recommended items to address in a clinical trial protocol and related documents*

| **Section/item** | **ItemNo** | **Description** |
| --- | --- | --- |
| **Administrative information** | | |
| Title | 1 | IMPROVE: Improving Recovery and Outcomes Every Day after the ICU |
| Trial registration | 2a | NCT03095417 Registered on March 23, 2017; last updated on May 15, 2017. |
|  | 2b | All items from the World Health Organization data set have been included in this form. |
| Protocol version | 3 | 7/5/2017 Version 1 |
| Funding | 4 | Funded by the National Institute on Aging (NIA) R01-AG055391. |
| Roles and responsibilities | 5a | Sophia Wang^1,2,3^, Jessica Hammes,^4^ Sikandar Khan^5^, Sujuan Gao^6^, Amanda Harrawood^7^, Stephanie Martinez^7^, Lyndsi Moser^1^, Anthony Perkins^2^, Frederick W. Unverzagt^1^, Daniel O. Clark^7,8^, Malaz Boustani^2,3,7,8^, Babar Khan^3,5,8^    ^1^Department of Psychiatry, Indiana University School of Medicine, Indianapolis, IN  ^2^Center of Health Innovation and Implementation Science, Center for Translational Science and Innovation, Indianapolis, IN  ^3^Sandra Eskenazi Center for Brain Care Innovation, Eskenazi Hospital, Indianapolis, IN  ^4^College of Arts and Sciences, Indiana University Bloomington, Bloomington, IN  ^5^Division of Pulmonary, Critical Care, Sleep and Occupational Medicine, Department of Medicine, Indiana University School of Medicine, Indianapolis, IN  ^6^Department of Biostatistics, Indiana University School of Medicine, Indianapolis, IN  ^7^IU Center of Aging Research, Regenstrief Institute, Indianapolis, IN  ^8^Division of Geriatrics and General Internal Medicine, Department of Internal Medicine, Indiana University School of Medicine, Indianapolis, IN |
|  | 5b | National Institute on Aging  R01AG055391  Program Officer Dr. Luci Roberts [roberlu@mail.nih.gov](mailto:roberlu@mail.nih.gov) |
|  | 5c | The study sponsor has no involvement in the design of this study or the drafting of this manuscript. |
|  | 5d | Dr. Babar Khan is the PI of the trial; he is a critical care scientist with expertise in ICU delirium and ICU acquired cognitive impairment. Dr Fred Unverzagt is a neuropsychologist with national expertise in Alzheimer’s disease and cognitive recovery; he will be a Co-I. Dr Dan Clark, a medical sociologist who works with the development and implementation of physical exercise programs, is another Co-I. The senior investigator is Dr. Malaz Boustani who is an expert in Alzheimer’s and aging research. Dr Sujuan Gao is another Co-I who has experience in designing statistical analytic plans for evaluating interventions targeting patients with delirium, MCI, and Alzheimer’s Disease. There will also be different sets of research teams in order to keep the study blinded; one team to recruit and take baseline assessments, one to randomize, one to carry out treatments, and one to collect follow-up data. |
| **Introduction** |  |  |
| Background and rationale | 6a | The purpose of this trial is to quantify the additive benefits, if any, of conducting cognitive training and physical exercise together in the recovery of ICU patients 50 and older who have experienced delirium during their ICU stay. Studies such as the SMART trail found benefits in brain function and repair in patients who did cognitive training after they were released from the ICU. The CARB trial found improvement in mental function with respect to memory problems for patients who had cognitive training and physical exercise sessions after being released from the ICU. This study did not however, use an attention control. The purpose of this trial is to compare the combined results to those of an attention control in order to prove the prediction that there are additive benefits in combing the two types of training. 70% of older adults are effected by delirium in the ICU; the presence of ICU delirium predisposes ICU survivors to long-term cognitive impairment and dementia. The correct and most effective post ICU care could in turn prevent or slow down the onset of Alzheimer’s disease and related dementias as these patients continue to age. There are no harms associated with the cognitive intervention, the cognitive control, or the physical control. The only harm associated with the physical intervention is over exertion; however, trained staff will be monitoring patients to ensure they do not over exert themselves. The benefit associated with physical exercise is induced neural proliferation. Cognitive training in turn promotes functional integration of these new neural elements into adaptive networks. |
|  | 6b | The chosen comparators are standards in this field that have been used before and have provided reliable results. Evidence was taken from previous studies to ensure accurate results from use. |
| Objectives | 7 | In comparison to older ICU survivors randomized to the attention control or either intervention alone, those randomized to 12 weeks of combined physical exercise and cognitive training will have higher total index cognitive scores as assessed by the Repeatable Battery for the Assessment of Neuropsychological Status (RBANS) at 3 and 6 months post randomization. In addition, those placed in this combined intervention group will have higher physical performance as measured by short physical performance battery (SPPB) and two-minute step test. Secondary results should be lower mood and anxiety symptoms as measured by Patient Health Questionnaire (PHQ-9) and Generalized Anxiety Disorder (GAD-7) scale, and higher quality of life as measured by the Medical Outcomes Study 36-item short form (SF-36) at 3 and 6-months post randomization. |
| Trial design | 8 | The proposed study is a four arm randomized controlled trial with an allocation ratio of 1:1 in which we hope to prove that the combination of physical activity and cognitive training is superior to a single intervention on its own. |
| **Methods: Participants, interventions, and outcomes** | | |
| Study setting | 9 | Patients for the study will be recruited from three Indiana University School of Medicine affiliated hospitals; IUH Methodist hospital, IUH University hospital; and Eskenazi Hospital. |
| Eligibility criteria | 10 | The inclusion criteria for the study is the following: patients aged ≥ 50 years, admitted to medical and/or surgical ICUs at Methodist, University, or Eskenazi hospitals, English-speaking, discharged home or subacute rehabilitation, able to provide consent or has a legally authorized representative to provide consent, access to a telephone, and have at least one episode of delirium as determined by the Confusion Assessment Method in the ICU. The exclusion Criteria is the following: self-reported diagnosis of cancer with short life expectancy, current chemotherapy or radiation therapy, history of dementing illnesses and other neurodegenerative diseases such as Alzheimer’s disease, Parkinson’s disease, or vascular dementia; current alcohol consumption ≥ 5 drinks per day, vision < 20/80 via Snellen card, low hearing or communicative ability that would interfere with interventions and outcome assessments, have delirium at the time of hospital discharge, have any American College of Sports Medicine absolute or relative contraindications to exercise, stroke as the admitting diagnosis, or a new event during the course of hospitalization; and recent history of drug abuse [Drug abuse and screening test (DAST-20) score>5]. |
| Interventions | 11a | Enrolled subjects randomized to the experimental intervention will receive cognitive training via computer-accessed online training modules; 45 minutes per session, 2 days per week for 3 months. Physical exercise will be delivered by trained facilitators to participants in their homes via internet-based single or multi-party (2-6 per group) videoconference; 45 minutes per session, 3 times per week for 3 months. The active cognitive intervention consists of a several modules from the Brain HQ developed by Posit Science Inc., that engage time-order judgment, visual discrimination, spatial-match, forward-span, instruction-following, dual task, and memory. Exercises adapt to participants’ level of performance and automatically advance in difficulty level as performance improves.  The cognitive control activity consists of control modules from the Brain HQ developed by Posit Science Inc. The cognitive control activities happen at the same frequency as the active cognitive intervention.    The active physical exercise intervention consists of 45 minutes of multi-modal physical exercise focused on seated aerobic and progressive resistance training designed to improve aerobic capacity, muscular strength and endurance consistent with current exercise recommendations. Three sessions per week for 3 months will be delivered. Participant’s heart rate or rating of perceived exertion (RPE) will be used to ascertain achievement of moderate intensity exercise. Participants will be advised to exercise at the RPE level of 5 to 6 on a 10-point scale. Generally, for adults over the age of 40 years, moderate-intensity is 5 to 6 on a 10-point exertion scale. Each physical exercise session will be divided as follows: 5 minutes of warm-up, 10 minutes of upper-body, 10 minutes of core, 10 minutes lower-body, 5 minutes of upper-body and lower-body, and 5 minutes of cool-down and flexibility exercises. All exercises will be conducted from a seated position in a solid backed chair.  The physical exercise control will consist of stretching and discussion of successful aging. Three sessions per week for 3 months will be delivered. The successful aging module was designed specifically as an attention control condition for exercise trials and has been used prior. Interventionists will deliver educational discussions on a variety of topics including nutrition and bone health. |
|  | 11b | We have incorporated exercise program elements that are associated with lower risks of cardiovascular complications and muscular injury. These include warming up, flexibility exercise, moderate intensity exercise, and a gradual progression of exercise intensity and duration. The Interventionist will educate subjects about the signs and symptoms of angina, myocardial infarction, and muscle/tendon related injuries and how to respond to them; a procedure recommended by the American College of Sports Medicine and the American Heart Association as an effective means of reducing complications to exercise. Cognitive training is relatively safe but anxiety and burden will be monitored at weekly intervals via participant reports on pop-up computer surveys.There will be a “step-down” battery if a participant is unable to tolerate the full outcome assessments, which will allow for an estimation of the treatment effect in key domains (Coding, SPPB and PHQ). |
|  | 11c | There are measures in place to ensure retention including availability of make-up sessions and availability of individual training sessions. Cognitive Intervention adherence data will be taken from Posit Science and if retention drops below 80%, the study staff will follow-up with the study participant to troubleshoot any technical issues and provide coaching. Physical training sessions will be offered throughout the week on varying days and times, including the weekends to provide ample training opportunities and make-up sessions. Gift cards will be used as incentives for training sessions and fair subject payments will be implemented for the completion of the 3 and 6 month follow-up outcome assessments. Assistive devices such as glasses, headphones, or pocket talkers will be provided if a participant’s visual or auditory acuity declines and interferes with interventions or outcome assessments. |
|  | 11d | There are no restrictions on participants during this trial. |
| Outcomes | 12 | At hospital discharge and baseline we will measure the subject’s age, race, gender, years of education, visual acuity, height, weight, body mass index, heart rate, blood pressure, Charlson Comorbidity Index, APACHE II score, activities and instrumental activities of daily living (ADL/IADL) prior to ICU admission through Katz and Lawton scales, cognitive status prior to admission through IQCODE, admission and discharge diagnoses, duration of mechanical ventilation, duration of delirium, ICU/hospital length of stay, and ICU and in-home medications. These measures will be used to describe the ICU survivors characteristics and as potential confounders. The Repeatable Battery for the Assessment of Neuropsychological Status (RBANS) total index score will provide the primary outcome for the trial. Secondary outcomes will be assessed using individual neuropsychological tests of processing speed, executive control, and new learning ability as follows: Trail Making Test Part B (seconds to complete), and Stroop Color and Word Test (interference trial). These measures sample major domains of cognition affected in ICU survivors. Physical training effects on balance and strength will be assessed via the Short Physical Performance Battery (SPPB), a validated objective assessment; grip strength will also be measured via the JAMAR hand dynamometer. The SPPB yields a performance score of 0-12 (0-4 poor, 5-7 intermediate, 8-12 good). A difference of 1 point indicates a significant change in function. Physical training effects on cardiovascular fitness will be assessed via the 2-minute step test. We have selected the 2-minute step test for cardiovascular fitness because it is a validated measure of aerobic capacity, does not require equipment, and can be used in the home setting. We will use the Patient Health Questionnaire–9 (PHQ9) and Generalized Anxiety Disorder Scale (GAD-7) to determine the impact of the intervention on ICU’s survivors’ mood and anxiety. The PHQ-9 is a nine-item depression scale with a total score from 0 to 27 and the GAD-7 is a seven-item anxiety scale with a total score from 0 to 21. Both of these scales are derived from the Patient Health Questionnaire, have good internal consistency, and test–retest reliability as well as convergent, construct, criterion, procedural and factorial validity for the diagnosis of major depression and general anxiety disorder. ICU survivors’ health–related quality of life will be assessed using the Medical Outcome Study Short Form (SF-36). This scale has eight components (physical functioning, role-physical, bodily pain, general health, vitality, social functioning, role-emotional, and mental health) that are aggregated into a Physical Component Summary (PCS) and a Mental Component Summary (MCS). We will use both the PCS and the MCS as the quality of life outcomes. Changes that differ between groups by 2 or more points on a scale of 0 to 100 have been shown to be clinically or socially meaningful. Blood samples will be collected and stored according to biological samples protocol to maximize sample quality and decrease sample |
| Participant Timeline | 13 | variability due to sample collection and processing techniques. Blood samples will be collected at baseline and at 3 months. The 3-month time-point is chosen because this marks the immediate post-training assessment phase, a point where we expect to see the maximum effect of interventions, improvements in the neurobiology and concomitant biomarker profile. 20 ml of blood will be collected from each patient. These tubes will then be labelled with pre–printed labels with a de-identified code. To avoid biomarker degradation and platelet activation, 18ml blood will be centrifuged immediately at 3000 x g. The supernatants will be removed and stored in 0.5ml aliquots so that multiple episodes of freezing and thawing will be unnecessary during assay procedures. 2ml fresh blood will be used for DNA extraction. The tubes will be placed on dry ice for transport. We will measure circulating levels of the pro-inflammatory cytokines (IL-1, 6, 8, TNF-α); the acute-phase reactant (CRP); neurotrophic factors (IGF-1, VEGF, BDNF); and markers of glial dysfunction and astrocyte activation (S-100β, GFAP). Serum marker quantifications will be performed in duplicates using pre-validated commercially available assay kits. Each kit employs 1-2 positive control(s) of known concentration and a negative control in every run. We will follow manufacturer provided established protocols for these assays. Genomic DNA will be extracted from blood samples using the DNeasy Blood & Tissue Kit (Qiagen, Inc., Valencia, CA) according to the manufacturer's protocol. Approximately 50ng of genomic DNA will be used for amplification. APOE genotypes will be determined by restriction enzyme digestion of amplified DNA. As number of recent studies have reported that a low ratio of plasma amyloid-β Aβ42 over Aβ40 is associated with increased Alzheimer’s disease risk and greater cognitive decline, we will explore the effect of a low ratio on our intervention. Samples will be analysed using standard analysis techniques. In addition to patient reported emergency department and hospital admission data, we will use the local data-warehouse to capture all of the data needed to determine utilization. Furthermore, we will also use the data from the Indiana Network for Patient Care (INPC) to complement any data use outside of our health system. INPC is the primary health information exchange in the state of Indiana and it provides data for acute care services from all of the health care systems within the state of Indiana. We will determine the number of emergency department visits and the number of re-hospitalizations during follow-up as well as the diagnoses associated with each utilization episode.  There will be a 3 month follow up for all patients. For the full timeline, see figure 3.  Time schedule of enrollment, interventions (including any run-ins and washouts), assessments, and visits for participants (see Figure 3). |
| Sample size | 14 | Cognitive training has been found to have a moderate effect size of approximately 0.5 SD and the combined training was found to have a larger effect size of 0.9 SD when compared to the control group in healthy elderly subjects.  Assuming effect sizes of 0.4 SD in the cognitive training only group and the exercise only groups at 3 and 6 month post baseline compared to the attention control group and effect size of 0.8 SD in the combined training group at 3 and 6 month post baseline compared to the attention group, a sample size of 60 patients in each group will yield 83% power at detecting a significant group by time interaction in a mixed effect model adjusting for correlations of 0.2 for outcomes measured 3 month apart and correlations of 0.1 for outcomes measured 6 month apart at α=0.05. The power estimation was conducted using the GLMPower procedure in SAS. To further assume that 30% patients may miss some post-baseline assessments, we will need to enroll a total of 344 patients into the study (86 patients per group). |
| Recruitment | 15 | Study personnel at each site will screen for eligible subjects each day using the ICU census. Eligible individuals (those who meet inclusion criteria and do not meet any exclusion criteria) will be screened twice per day for delirium until ICU discharge using the CAM-ICU. We have years of experience utilizing CAM-ICU in all our prior ICU studies. Patients who screened positive on the CAM-ICU and survived the ICU stay will be approached for enrollment into the study within 48 hours of their anticipated hospital discharge. Based on a delirium prevalence rate of 40%, a discharged home rate of 60%, a 30-day survival rate of 70%, and eligibility rate of 40% we project that there will be at least 280 potential subjects admitted to one of the 166 ICU beds across Eskenazi, Methodist or University hospitals every year. Assuming a consent rate of 50% and a recruitment period of three years and nine months, we will be able to have a pool of more than 500 subjects to meet the target sample size of 344. |
| **Methods: Assignment of interventions (for controlled trials)** | | |
| Allocation: |  |  |
| Sequence generation | 16a | Within 2 weeks of hospital discharge and after obtaining an informed consent, study staff will complete a baseline assessment consisting of measures of cognition, physical function, depression and anxiety, and quality of life. Blood samples will also be collected from the participants at the time of the baseline assessment. After initial assessment, study subjects will be randomized to study groups. Randomization will be stratified by age (50-64, 65-75, older than 75), discharge status (home or other), and study site. A computer-generated randomization within stratum will be done using random blocks of 4 or 8 (80% randomization block size 4:20% blocks block size 8). |
| Allocation concealment mechanism | 16b | Randomization will be stratified by age (50-64, 65-75, older than 75), discharge status (home or other), and study site. A computer-generated randomization within stratum will be done using random blocks of 4 or 8 (80% randomization block size 4:20% blocks block size 8). |
| Implementation | 16c | A computer-generated randomization within stratum using random blocks of 4 or 8 (as above) will be used to allocate patients to each arm of the study. Research assistants who are different than those conducting the follow up assessments will be the ones who enroll participants and assign them their interventions. |
| Blinding (masking) | 17a | The research assistants will be trained and blinded for the study. Multiple techniques will be used to ensure concealment of outcome assessments given by the assistants. Structured assessments that do not provide room for qualitative interviewing will be used to ensure unblinding. These assistants will not be involved in the study assignments or the treatment administrations. |
|  | 17b | Only the project manager and the primary investigator have access to the individual codes identifying the patients. |
| **Methods: Data collection, management, and analysis** | | |
| Data collection methods | 18a | Research assistants will complete the 3 and 6 month outcome assessments. Each test will be repeated to ensure quality and accuracy. The assessments will be the RBANS, PHQ-9, GAD-8, SPPB, 2 minute step test, grip strength, and SF-36. The data will be collected using Research Electronic Data Capture (REDCap). Multiple techniques will be used to ensure concealment of outcome assessments. |
|  | 18b | Monetary incentives will be provided to patients who complete both the 3 month and 6 month follow ups. At each follow up subjects will complete a RBAN, SPPB, a 2 minute step test, grip strength, PHQ-9, GAD-7, SF-36 PCS and a MCS assessment. Blood will also be drawn for testing the changes in the serum levels of CRP, IL-1, IL-6, IL-8, TNF- α, S-100β, GFAP, BDNF, VEGF, and IGF-1. |
| Data management | 19 | We will assure the privacy of subjects and confidentiality of the study data by assigning unique identifiers to track participant’s data instead of using their names, hospital, or social security numbers. All the records will be kept under lock with access granted only by study personal. The final data files for this study will be merged, maintained, and analysed on servers managed by the Division of Biostatistics, Department of Medicine, Indiana University School of Medicine. This group has extensive experience in the handling and security of PHI. None of the individual participants’ data will be identifiable in published reports or manuscripts; the analysable datasets will not contain the participant’s unique identifier. |
| Statistical methods | 20a | Statistical analyses will be conducted using SAS 9.4. Changes in patients’ baseline characteristics will be compared via ANCOVA for continues variables and the Cochran-Mantel-Hansel statistic for categorical variables while adjusting for age group. Mixed effects models will be used in addition to repeated RBANS scores (baseline, 3 months, 6 months) to collect data on the outcome measures. Post-hoc comparisons will be used to compare the effect of combined training verses the other three groups. Separate mixed effect models will be used for Trail Making Test Part A and B and Stroop Color and Word Test. |
|  | 20b | Analysis of the data will only be adjusted if one of the participants fails to be able to complete all aspects of the study. Specific measures will be taken to correlate incomplete data with the completed study data. |
|  | 20c | Missing data will be accounted for by the mixed effects model due to the assumption that the missing data will be at random. The baseline characteristics of patients with missing data will be compared to detect violations to the assumption. Sensitivity analyses will also be performed using imputation or a full parametric likelihood approach assuming various patterns of missing data. |
| **Methods: Monitoring** | | |
| Data monitoring | 21a | The study coordinator and biostatistician will generate reports for the PI, the safety officer, and the DSMB. These reports will contain a summary of adverse events and an explanation of how each event was handled, a summary of complaints and how each complain was handled, subject retention including the number of reasons of participant withdrawals, intervention compliance (session attendance), and a summary of protocol violations and how each was handled. All reports will be submitted to IU IRB for continued review. We plan to present unblinded adverse events data to the safety officer throughout the trial and to the DSMB panel when requested by the safety offer and at bi-annual meetings. If there is evidence of elevated adverse events, the safety officer will consult with the statistician and the PI. The PI will be notified within 24 hours of identification of any adverse events. Serious events will be reported within 5 business days to IU IRB, the Safety Officer, DCM, and NIH. In cases where there is any question regarding the level of adverse events or attributable cause, we will consult with DSMB and the safety officer. |
|  | 21b | All data will be de-identified prior to analysis. The principal investigator and project manager will have access to the individual code to identify individuals in the study. If an adverse event occurs, the individual will be identified and it will be up to the principal investigator and the project manager to terminate the study. |
| Harms | 22 | In the event of an adverse incident, the principal investigator and project manager will have access to the individual code to identify the individual involved. They will then update the status of the trial at ClinicalTrials.gov. |
| Auditing | 23 | The investigators will be a part of the process but the sponsor is independent. The DSMB will hold an initial meeting to approve the study and initiate recruitment, followed by a 6 month review, a 12 month review, and once every 12 months thereafter. |
| **Ethics and dissemination** | | |
| Research ethics approval | 24 | The protocol was already approved by the Institutional Review Board at the Indiana University School of Medicine. |
| Protocol amendments | 25 | Any changes in protocol or informed consent will be sent to the Institutional Review Board as protocol amendments so that they can be disseminated out to those involved and keeping track of the study. |
| Consent or assent | 26a | The principal investigator and the research staff are in charge of supplying and securing patient’s informed consent paperwork before enrolling them into the study. |
|  | 26b | Patients signed consent forms that provided all the information about the studies and what kind of biological samples will be taken. |
| Confidentiality | 27 | Efforts will be made to keep patients’ personal information confidential. The information will only be shared between people directly involved in the trial, the study sponsor, and (as allowed by law) state or federal agencies. Patient information will be stored securely and will not be shared with anyone outside of the study. |
| Declaration of interests | 28 | There are no conflicts of interest for this study. The agency who provided the funds had no role in the development of the study design, collection, analysis, interpretation of data, or the decision to submit the manuscript for publication. |
| Access to data | 29 | The only organizations who will have access to patients’ personal information are the study investigator and his research associates, the Indiana University Institutional Review Board or its designees, the study sponsor, National Institute on Aging, and (as allowed by law) state or federal agencies, specifically the Office for Human Research Protections (OHRP) and the National Institutes of Health (NIH). |
| Ancillary and post-trial care | 30 | In the event of injury stemming from participation in this study, the necessary medical treatment will be provided and billed as part of medical expenses. Costs not covered by the patient’s health care insurer will be the patient’s responsibility. There is no program in place for other monetary compensation for such injuries. However, by partaking in this trial, the patient is not giving up any legal rights or benefits to which they are otherwise entitled. |
| Dissemination policy | 31a | This trial will be published on Clinicaltrials.gov; any interested party will be able to view this study. |
|  | 31b | There is no intention of using professional writers on this trial. |
|  | 31c | The study will be available on Clinicaltrials.gov. |
| **Appendices** |  |  |
| Informed consent materials | 32 | Model consent form and other related documentation given to participants and authorised surrogates |
| Biological specimens | 33 | Plans for collection, laboratory evaluation, and storage of biological specimens for genetic or molecular analysis in the current trial and for future use in ancillary studies, if applicable. |

*It is strongly recommended that this checklist be read in conjunction with the SPIRIT 2013 Explanation & Elaboration for important clarification on the items. Amendments to the protocol should be tracked and dated. The SPIRIT checklist is copyrighted by the SPIRIT Group under the Creative Commons “[Attribution-NonCommercial-NoDerivs 3.0 Unported](http://www.creativecommons.org/licenses/by-nc-nd/3.0/)” license.
